# From diagnosis to therapy: the acute traumatic hemothorax – an orientation for young surgeons

**DOI:** 10.1515/iss-2023-0062

**Published:** 2024-02-16

**Authors:** Romina M. Rösch

**Affiliations:** Department of Thoracic Surgery, University Hospital Heidelberg, Heidelberg, Germany

**Keywords:** traumatic hemothorax, thoracic trauma, polytrauma management, eFAST, video-assisted thoracic surgery

## Abstract

**Introduction:**

This review aims to provide an overview of diagnosing and managing traumatic haemothorax for young surgeons.

**Content:**

Of 27,333 polytrauma patients in Germany in 2021, 35 % were admitted with thoracic trauma. In polytrauma patients, chest injuries are an independent negative predictor of 30-day mortality. These patients should be treated in an evidence-based and standardized manner to reduce mortality and morbidity. There are several methods of immediate diagnosis that should be used depending on hemodynamic stability. In addition to physical examination and chest X-ray, more specific techniques such as the eFAST protocol and Computed tomography (CT) of the chest  are available. Once the source of bleeding has been identified, acute treatment is given depending on hemodynamic stability. Thoracic drainage remains the gold standard in the initial management of hemothorax. If surgery is required because of an active source of bleeding, a hemothorax that has not been completely relieved, or associated injuries, either a minimally invasive or open approach can be used. The main focus is to stabilize the patient and avoid early and late complications.

**Summary and Outlook:**

Rapid and prompt diagnosis and management of traumatic hemothorax is essential for patient outcome and should be taught to all young surgeons who are in direct contact with these patients.

## Introduction

Hemothorax is defined as the accumulation of blood in the pleural space (space between the parietal and visceral pleura), typically characterized by a hematocrit level of ≥50 % [1]. However, hemothorax cannot be ruled out even with a pleural fluid hematocrit of less than <50 %, as dilution may occur if there is a delay in diagnosis and analysis of the pleural fluid. 

In such cases, hematocrit values of 25–50 % in pleural fluid are more common [2]. Therefore, a patient with suspected hemothorax and a pleural fluid hematocrit of less than 50 % should be further investigated for an active source of bleeding.

Examination of pleural fluid is important to differentiate between a hemothorax and a hemorrhagic pleural effusion [3].

Hemothorax can be classified according to different criteria. In addition to clinical relevance (stable vs. unstable patient), classification according to severity in relation to blood volume is crucial and, conversely, also decisive for clinical relevance.

A hemothorax with an intrathoracic collection of less than 400 mL is classified as a minimal hemothorax, 400–1,000 mL defines a moderate hemothorax and >1,000 mL a massive hemothorax [4].

Each hemothorax can contain 40 % of a patient’s circulating blood volume, so in addition to the symptoms of haemorrhagic shock, underlying medical conditions such as coronary artery disease may be present.

Therefore, prompt diagnosis and treatment are critical for patient’s outcome. In most cases, if patients are treated in time, they can have a good outcome without suffering long-term side effects, depending on the associated injuries.

## Epidemiology and etiology

Hemothorax is one of the most common conditions seen in trauma and thoracic surgery. Trauma is the term used to describe an external event that injures or damages an organism, or the resulting damage itself. The term injury is used synonymously.

Exact data on the incidence of traumatic hemothorax in Germany are not available, but the annual report of the Trauma Registry of the DGU (“Deutsche Gesellschaft für Unfallchirurgie”) showed that there was a total of 27,333 polytrauma patients in 2021.

In total, 35 % of the 27,333 accident victims suffered thoracic trauma. Overall, in 2021, 2.5 % of casualties were treated at the scene and 9 % of the 27,333 patients received a chest tube in the trauma room. That’s a total of 3,143 patients with chest tubes [5].

Causes of hemothorax vary, including spontaneous, iatrogenic and traumatic. Thoracic trauma is the most common cause [4, 6], [7], [8], followed by iatrogenic causes (e.g. from biopsy punctures, pleural effusion drainage) [9] and spontaneous causes like pneumthorax, hemophilia, aneurysms, intrathoracic malignancies and others [2, 6].

Traffic accidents are the most frequent cause of trauma (45 %), followed by falls from a low height (≤3 m) with 27.9 %, falls from a high height (>3 m) with 15.6 %, suicides with 4.6 % and crimes with 2.4 % [5].

The source of bleeding is often localized in the chest wall, lung parenchyma, heart or great vessels, and the causes may be traumatic or non-traumatic. In case of non-traumatic hemothorax, causes include thoracic malignancy, anticoagulant medication, coagulopathies, aortic dissection, tuberculosis or necrotising infection. Traumatic injury, including chest trauma with hemothorax, is one of the leading causes of death in people under the age of 45 [10].

## Diagnostic procedures

The majority of hemothorax are traumatic in origin, as described above, there is a high mortality rate due to hypovolemic shock, so it is crucial to make the diagnosis as soon as possible in order to start treatment immediately.

Therefore, the optimal diagnosis should be rapid, accurate and non-invasive. In addition, computed tomography is the gold standard for diagnosis, but is complicated by time delay and transport from the emergency department especially in unstable patients [11]. If a whole-body computer tomographie (CT) including cranial CT is not available, at least an eFAST scan should be performed to evaluate for free fluid [12].

## (e)FAST (Extended-Focused Assessment with Sonography in Trauma)

The introduction of point-of-care ultrasound has had a significant impact on patient assessment and management. Ultrasound has significant advantages, including bedside availability, ease of use and reproducibility. In addition, this form of diagnosis is non-invasive with no radiation exposure and can be used without hesitation in renal insufficiency and contrast agent allergy. Sonography has been used in the diagnosis of intraperitoneal free fluid since the 1970s and in the USA and Europe since the 1990s [11].

FAST is an ultrasound protocol developed to detect haemoperitoneum and haemopericardium. Numerous studies have confirmed its effectiveness, including in trauma patients [13].

As with all tests, the practice of the physician performing the FAST protocol is critical. In the United States, 96 % of level 1 trauma centers routinely perform this diagnostic procedure as part of Advanced Trauma Life Support (ATLS) [14].

### So what is the role of the eFAST protocol in hemothorax?

The FAST protocol was implemented in trauma units in 1996 as part of the expanded ATLS guidelines. More recently, the extended FAST protocol (eFAST) was incorporated into the trauma algorithm, adding hemothorax and pneumothorax to the diagnostic spectrum. Focused ultrasound is used to assess for free fluid in the pleural, pericardial or (abdominal) cavities and to exclude or confirm a pneumothorax.

The eFAST has become firmly established in trauma management in the shock room and has found its way into ATLS. It is designed to speed up diagnosis so that potentially lethal injuries can be identified and treated in the emergency room. In the so-called “ABCDE” scheme, ultrasound is assigned to “B-breathing” and “C-circulation and haemorrhage control” [12].

In the revised S3 guideline for the treatment of polytrauma/severe injury, ultrasound has found its way into the recommendations in several areas [15, 12].

The indications for the eFAST protocol include all trauma patients with circulatory instability or impaired consciousness. These patients are often in a time-critical situation, and the experienced physician can perform the protocol in less than 2 min to provide an initial assessment. Another advantage, in addition to its wide availability, is that it can be used simultaneously with other vital measures in the emergency room [12].

Ultrasound has consistently been shown to be more sensitive than chest radiography in detecting hemothorax. As radiography is known to have its limitations, a total of 300–500 mL of intrathoracic blood is required for adequate detection on chest radiography [16]. In supine patients, up to 1,000 mL of blood are required to avoid being overlooked on X-ray [16]. It is well known that polytrauma patients are admitted to the trauma unit in the supine position. In contrast to chest X-ray, ultrasound can detect even smaller volumes of about 100 mL [17].

Despite the increasing use of ultrasound in the diagnosis of traumatic injury, there are limitations. Accuracy is highly dependent on the experience of the clinician [18] and despite its high sensitivity, computed tomography is more sensitive and provides more findings than sonography [19].

## Computed tomography

Computed tomography (CT) of the chest  is frequently used to identify additional injuries and to help localize sources of bleeding after bedside chest radiography and ultrasound [20].

Once the patient is hemodynamically stable, chest CT can be performed, preferably with intravenous contrast agent. CT can identify additional injuries in 20–30 % of patients with an initially abnormal chest radiograph. In the presence of a hemothorax, CT may show a collection of blood not seen on the chest radiograph [20]. Therefore, CT remains the gold standard for the diagnosis of hemothorax in the trauma patients.

## Treatment

In a large cohort of patients, Ekpe et al. showed that a delay of more than 24 h to the nearest hospital was associated with a significantly increased mortality rate (62.5 %). Patients admitted within the first 24 h had a mortality rate of 2.7 % in the study by Ekpe and colleagues [21].

**Figure 1:**
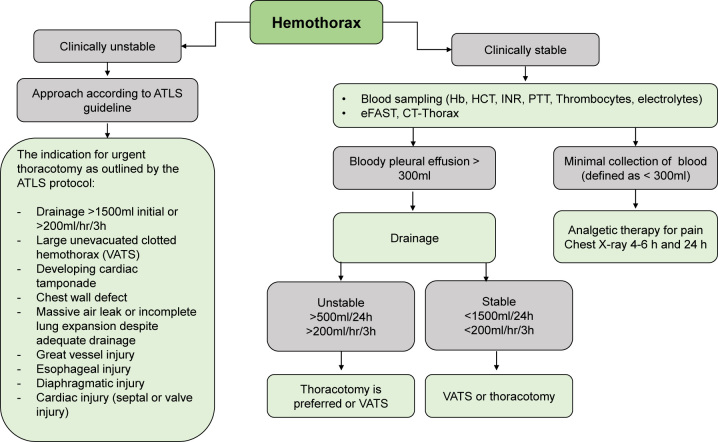
Treatment algorithm for hemothorax (from Lecturio) [22].

Life-threatening injuries need to be treated promptly, either by thoracotomy or, in severe cases, by sternotomy. The appropriate management of a patient with suspected hemothorax depends on their hemodynamic status and the rate of chest tube output, as shown in Figure 1 [22] below.

## Conservative approach

Conservative management is based on the overall severity of the chest injury and/or the amount of blood loss. If the patient is hemodynamically stable and the hemothorax is less than 300 mL, conservative management with observation, lung protective ventilation and pain control may be attempted [23].

If surveillance is attempted, it is recommended to check hemoglobin regularly as well as frequently repeated imaging (chest X-ray), especially in the first 24 hours to ensure stability of the hemothorax and to identify other potential pathologies [23, 24].

## Chest drainage

Chest drainage is the linchpin in the management of hemothorax. Not only is it frequently indicated for initial stabilization in the trauma unit, but also as a second diagnostic tool. For example, the pleural fluid can be analyzed and tested for hematocrit to classify hemothorax.

It has been shown that blood volume >300 mL increase the likelihood of requiring a chest tube up to four times. Patients with a hemothorax (volume <300 mL) had a 72–92 % chance of resolving without complications or intervention [25].

Bauman et al. showed in a randomized trial that in the stable patient with traumatic hemothorax, 14 French drains can be as effective as 28–32 French drains, with no significant difference in outcome. In the unstable patient requiring emergency drainage, a large chest tube drainage of 24–32 French is preferred [26].

This again shows that two factors play an important role in the management of hemothorax: the hemodynamic stability of the patient and the amount of intrathoracic blood volume.

## Fibrinolytic therapy

The ability of blood to coagulate has clear advantages and disadvantages in hemothorax. We need coagulation to stop active bleeding, but in the case of a coagulothorax, relief by drainage may be more difficult. In the inoperable patient, special therapy is required in such cases.

Several studies have shown that instillation of intrapleural fibrinolytic therapy (IPTF) into the retained hemothorax to dissolve blood clots and accelerate resolution and drainage by percutaneous drainage can be an effective method to avoid surgical intervention in high risk patients [27, 28].

## Surgical management

When the size, severity or associated injuries of a hemothorax warrant intervention, surgical management remains the method of choice. Most cases of hemothorax can be managed using video-assisted thoracic surgery (VATS). If blood remains in the pleural cavity after thoracoscopy, this is known as a retained hemothorax, which is associated with a significant risk of developing late complications such as empyema and fibrothorax. Once late complications occur, morbidity and mortality increase dramatically and the only definitive treatment might be reoperation [29].

## Video-assisted thoracic surgery

It is well known that VATS has lower morbidity, better visualization and a higher detection rate of small injuries with less postoperative pain compared to thoracotomy.

According to research from the DGU Trauma Registry, 0.9 % of trauma patients with an Injury Severity Score (ISS) ≥9 undergo emergency thoracotomy within the first hour of arrival at the hospital. During the entire hospital stay, 1.8 % of trauma patients require thoracotomy [30].

This implies that approximately half of the patients receive delayed surgical treatment and could potentially be managed by VATS rather than thoracotomy. Prospective studies on the use of VATS for the management of thoracic trauma are rare and the evidence is limited.

Furthermore, the recommendation for more than 50 years to perform thoracotomy in trauma patients with an initial blood loss ≥1,500 mL via the inserted chest tube or with a continuous blood loss ≥250 mL/h over 4 h is only relative to today’s better stabilization techniques [31].

In unstable patients with traumatic hemothorax unable to be stabilized acutely, prompt and urgent care is required. Thoracotomy often remains the method of choice, while VATS is recommended for a wide range of indications in the management of stable patients with thoracic trauma and hemothorax. Indications for VATS include persistent hemothorax, treatment of injuries and hemorrhage of the lung, diaphragm, chest wall and other organs, and in the secondary phase, treatment of thoracic sequelae of injury (empyema, persistent pulmonary fistula, infections or atelectasis, etc.) [31].

It is undisputed that the unstable patient with relevant thoracic trauma should undergo primary thoracotomy for rapid and immediate control of intrathoracic injuries.

A thoracotomy, sternotomy or even a clamshell thoracotomy is a relevant additional trauma for the polytrauma patient and should be well considered beforehand [32].

Schreyer et al. [31] provided good guidelines for the indication of VATS in traumatic hemothorax in their review 2023:

– VATS should only be used in patients who are respiratory and circulatory stable or stabilized [31].
– In stable patients with penetrating chest trauma, VATS can be used for diagnostic and therapeutic purposes [31].
– In a hemodynamically stable patient with hemothorax, VATS may be performed even in the presence of intra-thoracic hemorrhage, provided that there are no compelling reasons for thoracotomy or vascular intervention on CT [31].
– If there is a relevant retained hemothorax (≥300 mL), early VATS should be performed to relieve the hemothorax within the first 4 days after trauma [31].
– In the case of rib fracture with retained hemothorax (≥300 mL) and/or suspected relevant associated injury, synchronous VATS should be performed as part of planned thoracic wall osteosynthesis [31].

## Thoracotomy

Overall, regarding the indications for thoracotomy in traumatic hemothorax, the literature in recent years is quite clear but evidence is scarce. VATS is increasingly becoming the focus of care – in simple terms, any patient with an indication for surgical management of a traumatic hemothorax should receive VATS, and if this is not indicated based on the above criteria, thoracotomy should be considered.

If an emergency thoracotomy is required and the above criteria preclude VATS, the standard approach is the anterolateral approach, which provides excellent visualization of the thoracic cavity and especially the hilum [33]. In some types of injury, the anterolateral approach may not be sufficient. In this case, either the clamshell or hemiclamshell approach can be used [34]. The extended approaches should be considered as a team prior to implementation due to the greatly increased corridor damage.

The main indication for emergency thoracotomy is active bleeding in an unstable patient [35]. Certain internal injuries may also be indications for immediate thoracotomy, including injuries to the esophagus, diaphragm, heart and great vessels (e.g. aorta).

## Summary

In polytrauma patients, thoracic injuries are an independent negative predictor of 30-day mortality [36]. Of 27,333 polytrauma patients in Germany in 2021, 35 % were admitted with thoracic trauma. These thoracic trauma patients should be managed in an evidence-based and standardized manner to reduce mortality and morbidity.

Several techniques are available for immediate diagnosis and should be used depending on hemodynamic stability. For example, in addition to physical examination, chest radiography in the shock room is the basic diagnostic procedure. In an unstable patient, the trained examiner can assess both a hemothorax and a pneumothorax within a few minutes using the eFAST protocol with a high degree of reliability. Any stable or stabilized patient should receive a computed tomography scan to localize the source of bleeding and identify associated injuries.

Acute management of hemothorax has remained relatively stable over the decades. Chest tube drainage has been the gold standard in the initial management of hemothorax almost since its inception. In severe or complicated hemothorax thoracotomy has often been, and still is, the last resort in hemodynamically unstable patients or those with significant co-injuries. In hemodynamically stable patients, VATS has increasingly become the preferred procedure when surgery is indicated.

Prevention of residual hemothorax reduces the incidence of late complications such as empyema and fibrothorax, thereby improving overall morbidity and mortality. In addition to surgical management, intrapleural instillation of fibrinolytics offers a good alternative for residual hemothorax in this setting. A major advantage of fibrinolytics, in addition to reducing late complications, is the reduction of invasive surgical procedures, especially in the multimorbid patient.
